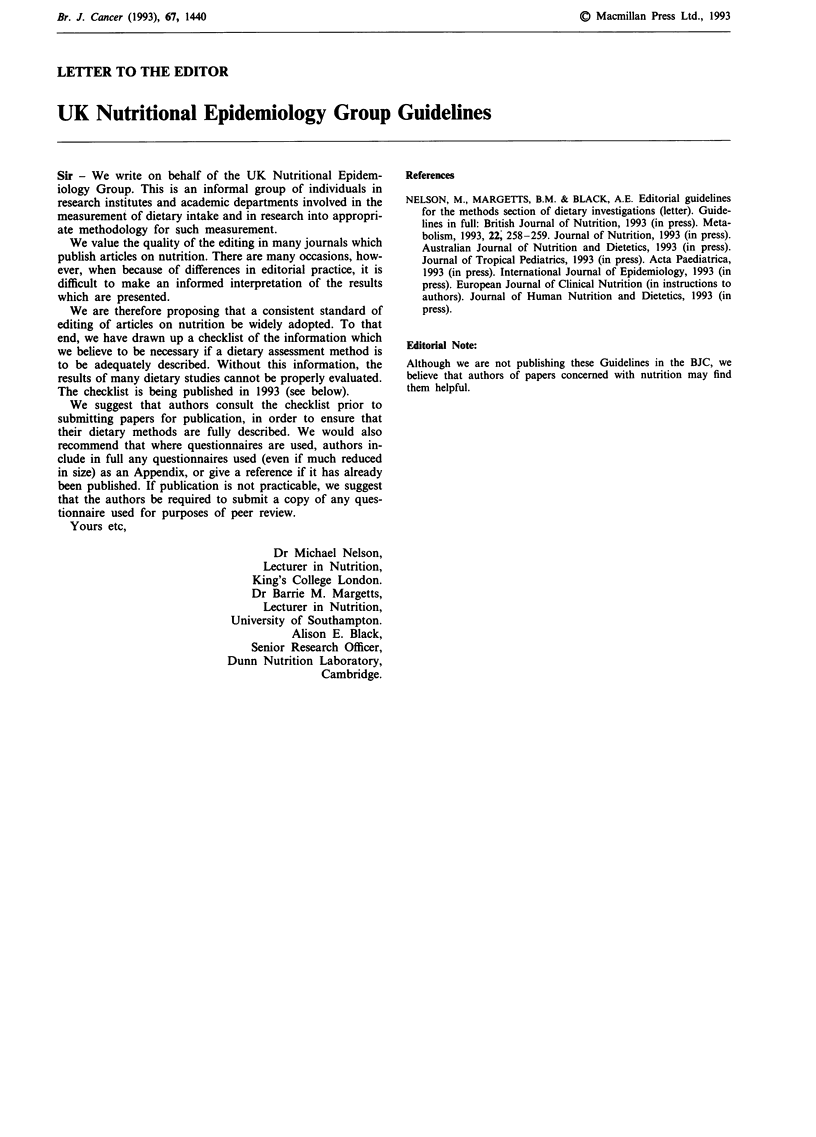# UK Nutritional Epidemiology Group guidelines.

**DOI:** 10.1038/bjc.1993.267

**Published:** 1993-06

**Authors:** M. Nelson, B. M. Margetts, A. E. Black

**Affiliations:** King's College, London, England.


 © Macmillan Press Ltd., 1993

LETTER TO THE EDITOR

### UK Nutritional Epidemiology Group Guidelines

**Sir** – We write on behalf of the UK Nutritional Epidemiology Group. This is an informal group of individuals in research institutes and academic departments involved in the measurement of dietary intake and in research into appropriate methodology for such measurement.

We value the quality of the editing in many journals which publish articles on nutrition. There are many occasions, however, when because of differences in editorial practice, it is difficult to make an informed interpretation of the results which are presented.

We are therefore proposing that a consistent standard of editing of articles on nutrition be widely adopted. To that end, we have drawn up a checklist of the information which we believe to be necessary if a dietary assessment method is to be adequately described. Without this information, the results of many dietary studies cannot be properly evaluated. The checklist is being published in 1993 (see below).

We suggest that authors consult the checklist prior to submitting papers for publication, in order to ensure that their dietary methods are fully described. We would also recommend that where questionnaires are used, authors include in full any questionnaires used (even if much reduced in size) as an Appendix, or give a reference if it has already been published. If publication is not practicable, we suggest that the authors be required to submit a copy of any questionnaire used for purposes of peer review.

Yours etc,

Dr Michael Nelson,
Lecturer in Nutrition,
King's College London.
Dr Barrie M. Margetts,
Lecturer in Nutrition,
University of Southampton.
Alison E. Black,
Senior Research Officer,
Dunn Nutrition Laboratory,
Cambridge.

**References**

NELSON, M., MARGETTS, B.M. & BLACK, A.E. Editorial guidelines for the methods section of dietary investigations (letter). Guidelines in full: British Journal of Nutrition, 1993 (in press). Metabolism, 1993, 22, 258–259. Journal of Nutrition, 1993 (in press). Australian Journal of Nutrition and Dietetics, 1993 (in press). Journal of Tropical Pediatrics, 1993 (in press). Acta Paediatrica, 1993 (in press). International Journal of Epidemiology, 1993 (in press). European Journal of Clinical Nutrition (in instructions to authors). Journal of Human Nutrition and Dietetics, 1993 (in press).

**Editorial Note:**

Although we are not publishing these Guidelines in the BJC, we believe that authors of papers concerned with nutrition may find them helpful.